# Elucidating the Common Generalist Predators of *Conotrachelus nenuphar* (Herbst) (Coleoptera: Curculionidae) in an Organic Apple Orchard Using Molecular Gut-Content Analysis

**DOI:** 10.3390/insects7030029

**Published:** 2016-06-24

**Authors:** Jason M. Schmidt, Zsofia Szendrei, Matthew Grieshop

**Affiliations:** 1 Department of Entomology, Michigan State University, 578 Wilson Rd., East Lansing, MI 48824, USA; szendrei@msu.edu (Z.S.); grieshop@msu.edu (M.G.); 2 Department of Entomology, University of Georgia, 2360 Rainwater Rd., Tifton, GA 31793, USA

**Keywords:** apple production, biological control, generalist predators, MGCA, natural enemies, plum curculio, organic, spiders

## Abstract

*Conotrachelus nenuphar* (Herbst) (Coleoptera: Curculionidae), plum curculio, is a serious direct pest of North American tree fruit including, apples, cherries, peaches and plums. Historically, organophosphate insecticides were used for control, but this tool is no longer registered for use in tree fruit. In addition, few organically approved insecticides are available for organic pest control and none have proven efficacy as this time. Therefore, promoting biological control in these systems is the next step, however, little is known about the biological control pathways in this system and how these are influenced by current mechanical and cultural practices required in organic systems. We used molecular gut-content analysis for testing field caught predators for feeding on plum curculio. During the study we monitored populations of plum curculio and the predator community in a production organic apple orchard. Predator populations varied over the season and contained a diverse assemblage of spiders and beetles. A total of 8% of all predators (eight Araneae, two Hemiptera, and six Coleoptera species) assayed for plum curculio predation were observed positive for the presence of plum curculio DNA in their guts, indicating that these species fed on plum curculio prior to collection Results indicate a number of biological control agents exist for this pest and this requires further study in relation to cultural practices.

## 1. Introduction

*Conotrachelus nenuphar* (Herbst) (Coleoptera: Curculionidae), the plum curculio, is a serious direct pest of Eastern United States tree fruit including, apples, cherries, peaches and plums. Damage is caused by oviposition in immature fruit and in later summer by adult feeding on ripening fruit. Oviposition consists of females cutting a crescent shaped flap in fruit skin prior to egg deposition in the resulting cavity [1]. Fruit containing larvae typically abscess from the tree and fall to the orchard soil surface. The fourth instar exits the fruit to pupate in the soil and adults typically emerge within 30 days [2]. In the northeastern USA, plum curculio have a single generation per year and it is primarily a problem in apple and cherry production, whereas plum curculio in the southeastern USA have two generations per year and are a significant pest of peaches [3].

Until recently, organophosphate insecticides have been the primary control tactic for plum curculio in tree fruit production. A variety of newer insecticide chemistries including insect growth regulators, neonicotinoids, and oxadazines are currently being used as alternative chemical tactics [4,5], but these are not registered for organic pest management. Thus, there is an increasing need for alternative sustainable plum curculio management tactics. Prior to the introduction of synthetic insecticides for conventional production, plum curculio were managed through cultural and mechanical tactics [6]. In addition, biological control has also been explored with several parasitoids identified but reported parasitism rates are typically below 5% [7,8].

Predators can also provide biological control services and generalist predators have been shown to be important for other pests in apple orchard systems [9,10,11,12,13,14,15]. Predation by generalist predators has been evaluated in the Southeastern USA and sentinel larvae removal rates of up to 62% have been reported with ants (Formicidae) identified as the primary predators [16]. However, the extent and importance of general plum curculio biological control has been extremely difficult to quantify due to the cryptic feeding location of larval insects, pupation in the soil and well camouflaged adults. Determining the identity of likely natural enemies of plum curculio is an important first step in evaluating the impact of generalist natural enemies of this pest. Historically, identifying predators has been accomplished through direct observations of predator prey interactions. Direct observations of plum curculio predation events have been difficult to obtain, and have relied on the use of sentinel prey [16]. Modern molecular techniques for the identification of predator gut contents, however, provide an elegant approach towards answering this question.

Molecular biology has revolutionized how we study and understand biological systems, allowing us to detect and differentiate specific organisms with much greater specificity than ever before. In the case of pest management we can now use molecular techniques to detect specific species when only partial samples are available and differentiate among populations on the basis of specific traits—e.g., pesticide resistance, physiology and even behavior [17,18]. Another recent application of molecular tools is the identification of predator food webs—allowing us to determine the function of specific predators in relation to key pests [19,20]. However, before any of these possibilities can be explored for plum curculio a DNA-based “toolkit” needs to be developed.

In this study we developed a DNA-based detection system for identification of plum curculio in predator gut contents (i.e., molecular gut-content analysis—MGCA) and evaluated field caught generalist predators at an organic apple orchard for evidence of feeding on plum curculio. The underlying rationale and approach of molecular gut-content analysis (MGCA) used here followed widely published protocols for studying predation in insect and spider predators (e.g., [21,22,23]). The overall aim of this study was to identify predators of plum curculio. In combination with the initial test of predation on plum curculio in Michigan organic apple production, we monitored populations of the plum curculio and the predator community.

## 2. Experimental Section

### 2.1. Description of Field Site

The experiment was conducted in an organic orchard system at Almar Orchards, Flushing, MI, USA. One-acre plots were established by separating sample locations within the fields by a minimum of seven rows of apple trees. The plots consisted of nine one-acre plots in a Honey Crisp orchard and six one-acre plots in a Red Delicious orchard for a total of 15 sample plots. Management of the orchards was standard organic practices for Michigan organic apple production throughout the season.

### 2.2. Pest Activity Sampling

Plum curculio pheromone lure traps (Great Lakes IPM, Vestaburg, MI, USA) that attract both males and females were placed on the edges of each field. Lures were replaced monthly. Traps were checked weekly for activity of plum curculio adults entering the fields. A second method to estimate plum curculio activity in fields was to monitor their damage to fruit in the plots as an indication of plum curculio pressure in the fields. To monitor damage, later in the season, fruit was visually assessed for presence of plum curculio oviposition stings. Four fruit per tree for 20 trees per plot of the 15 plots were assessed for oviposition damage.

### 2.3. Predator Population Sampling

Vacuum suction samples (using a modified leaf blower, Troy-Bilt 25cc Gas Blower/Vac, Troy-Bilt LLC, Cleveland, OH, USA) were collected to estimate predator abundance of broad taxonomic groups of commonly occurring arthropod predators. On five sampling dates over the 2014 growing season, suction samples were taken in plots at three random locations in the center of the plot. This provided a total of 225 suction samples. Each suction sample consisted of suction sampling for 30 s to remove all material within a Daubenmire frame (0.1 m^2^). These were transferred to plastic bags and taken to the laboratory for further processing. Berlese funnels were used to draw insects out of suction sampled leaf material and debris into vials containing 70% ethanol. Following Berlese, predatory beetles, hemiptera, and spiders were counted to estimate predator abundance. The three samples were pooled to form an estimate of predator population abundance in 0.3 m^2^ area of the plots.

### 2.4. Molecular Gut-Content Analysis

*DNA extraction and PCR*. Total DNA was extracted from individual whole arthropods using QIAGEN DNEasy Blood & Tissue Kits following the manufacturer’s animal tissue protocol (QIAGEN Inc., Chatsworth, California, USA). PCR (12 μL) consisted of 2× PCRBIO HS Taq Red Mix (PCR Biosystems Ltd., London, UK), 0.2 μM of each primer, and template DNA (1 μL of total DNA). PCRs were conducted on Mastercycler Pro (Eppendorf, Hamburg, Germany). Electrophoresis confirmed amplification using 10 μL of PCR product in 2% SeaKem agarose (Lonza, Rockland, ME, USA) stained with GelRed (0.1 mg/μL; Biotium Inc., Hayward, CA, USA). Reactions that did not show amplification were screened using cytochrome *c* oxidase subunit I (COI) primers LCO-1490 [24] and HCO-700ME [25] for arthropods to confirm the presence of amplifiable DNA in samples.

Primers targeting plum curculio were designed to test for predation on this pest by the predator community present in an organic orchard system. Commonly used protocols for designing primers for molecular gut-content analyses were employed [26]. *Conotrachelus nenuphar* specific primer pairs first selected using sequences EU522677, KF150699 in combination with the NCBI Primer-BLAST tool to look at possible primer pairs and specificity was tested against the total library for Arthropoda. A subset of Curculionidae COI sequences available on GenBank were aligned with the complete COI region of *Hylobitelus xiaoi* (NC022680.1) using MUSCLE [27]. After initial testing for specific primer pairs in *Primer 3* [28], and test conducted using Primer-BLAST, one primer pair was selected (Forward: PC42F: 5′-TCATCGGTCAAGAAAGAGGGA-3′ , T_m_ = 62 °C; Reverse: PC182R: 5′-TGCCCGTGTATCAACGTCTA-3′, T_m_ = 60 °C) yielding an amplicon size of 140 bp. The PCR reagents used to optimize the temperature settings were the same as above. The optimal PCR thermal cycling protocol was 95 °C for 2 min, followed by 34 cycles of 95 °C for 30 s, 54 °C for 45 s, and 72 °C for 15 s, and a final extension time of 5 min at 72 °C. In the laboratory, the primers were screened for cross-reactivity against 100 non-target arthropod DNA extractions including common species collected at the field site within the: (1) Araneae families: Araneidae, Corinnidae, Gnaphosidae, Linyphiidae, Lycosidae, Salticidae, Tetragnathidae, Thomisidae; (2) Coleoptera families: Carabidae, Chrysomelidae, Coccinellidae, Curculionidae (Curculioninae, *Tournotaris, Acoptus, Brachyderini, Stenopelmus*), Pyrochroidae, Staphylinidae, Tenebrionidae; (3) Collembola familes: Entomobryidae, Isotomidae, Sminthuridae; (4) Diptera families: Chironomidae, Culicidae, Drosophilidae, Muscidae, Syrphidae, Tabanidae, Dolichopodidae; (5) Hemiptera families: Aleyrodidae, Cercopidae, Cicadellidae, Cicadellidae, Delphacidae, Gerridae, Miridae, Psyllidae; (6) Hymenoptera families: Brachonidae, Pteromalidae, Pteromalidae; (7) Lepidoptera families: Arctiidae, Geometridae, Lasiocampidae, Noctuidae, Plutellidae, Tortricidae; (8) Neuroptera family Chrysopidae; (9) Opiliones; (10) Orthoptera families: Acrididae, Tettigoniidae; (11) Plecoptera family, Perlidae; and (12) Pseudoscorpionidae.

Predators were hand collected from the experimental plots on two dates (28 May 2014, and 2 June 2014) to test for predation. These dates were chosen to maximize the likelihood of interaction because peak populations of plum curculio are observed in mid-May to June [6]. Predators were collected by aspirator from the center of each plot, total of 15 one-acre plots, in three randomly chosen locations. Locations were at minimum 10 m apart. At each location, predators were collected from the ground by searching the area and collecting all predators. To collect predators from apple trees, a drop cloth was placed beneath the three randomly selected apple trees where vegetation and the branches above were beat to dislodge any predators from the vegetation. Each predator was immediately aspirated from the drop-cloth and transferred into a pre-chilled 1.5 mL microcentrifuge tube containing chilled 70% ethanol. Tubes from each plot were held on ice and once back at the laboratory, stored in a −20 °C freezer until extraction and PCR. Extraction, PCR and gel electrophoresis on field-collected predators followed the abovementioned protocols to identify plum curculio DNA in predator guts.

### 2.5. Data Analysis

To assess the change in predator abundance (pooled predator abundance across taxa) over time, we used Generalized Least Squares (gls) models that accounted for autocorrelation in time using the package “nlme{gls}” (i.e., repeated measures [29,30]. The proportion of predators positive for plum curculio predation was reported as descriptive statistics using the package “doBy{summaryBy}”. All statistical comparisons and figure generation were conducted in R version 3.2.0 [30].

## 3. Results

### 3.1. Pest Activity

Pheromone traps showed that plum curculio populations were low in this year of study, as we observed less than 20 plum curculio over the entire season across all the orchards. Trap data provides evidence that this elusive pest was present in the fields. In addition, we evaluated 6768 apples across the orchard within the 15 acre plots and found 650 to be damaged by oviposition scars, which provides further evidence of active populations in the orchard with approximately 9.6% of fruit damaged by plum curculio.

### 3.2. Predator Populations

**Figure 1:**
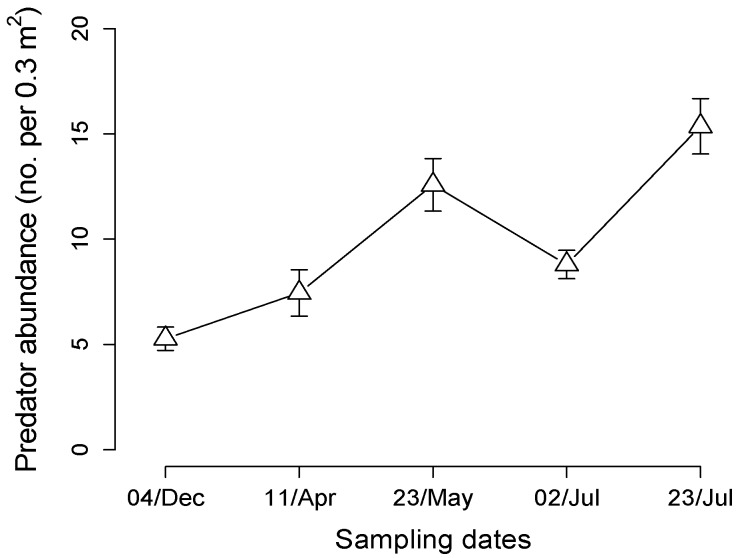
Predator abundance in field plots across the growing season. Predators were sampled within the center of one-acre plots using suction sampling of area within three placements of Daubenmire frame. Symbol represents mean abundance of all predator groups pooled over 15 plots for each date (bar indicates ± 1SEM).

We collected a total of 1058 predators in suction samples from 15 one-acre plots in an organic apple orchard system (Figure 1). Predators collected included Araneae, Carabidae, predatory Hemiptera, Opiliones, and Centipedes, and we pooled the samples to estimate overall predator population size and fluctuation over time. Predator abundance was significantly related to sampling date, and an indication that populations were increasing over the season (*F*_4,220_ = 9.26, *p* < 0.0001, Figure 1).

### 3.3. Molecular Gut-Content Analysis

The primer pair was optimized and elicited no non-target amplifications when tested against 100 arthropod DNA extractions. Our diverse database of non-targets used in this optimization step included 12 orders and 44 families within Arachnida and Insecta (see Methods for list of taxa).

On two sampling dates within the organic orchard system we were able to hand collect a total of 437 predators (Table 1). Ground and canopy sampling yielded at minimum 46 species including a diverse range of Araneae and Coleoptera all of which were PCR-assayed using plum curculio specific primers (Table 1). Over 20 spider species were collected and tested as were a minimum of 12 species of beetles (Table 1). These are conservative estimates because immatures and sub-adult spiders can only be identified to family or to genus.

Of the 437 total predators tested, 8.17% were positive for plum curculio (Table 2). This included a diversity of Araneae taxa (true spiders), and Coleoptera (beetles). Eight different spider species tested positive for feeding on plum curculio. Two common hemipteran natural enemies, *Nabis* sp. and *Podisus* tested positive, and six different coleopteran species (Table 2). The most frequently collected group was the Lycosidae spiders, a common agrobiont family in many agricultural systems. However, only one individual from this taxonomic group tested positive for feeding on plum curculio (Table 1).

## 4. Discussion

Our findings provide the first direct evidence that a variety of generalist predators feed on plum curculio in Michigan apple orchards. Organically managed orchards rely on biological control as an important pest management tool and our results provide the initial steps for designing a biocontrol program for plum curculio. Natural biological control in apple orchards has been demonstrated previously, for example in France, spiders and carabids feed on two economically important pests, the oriental fruit moth, *Grapholita molesta*, and codling moth, *Cydia pomenella* [9], two cosmopolitan species. Furthermore, in the south United States, Jenkins, et al. [16] observed increased control of plum curculio when natural enemies were present. Predation studies in the field and the laboratory provide evidence that biocontrol is an important component of pest management for orchard systems [16]. In addition, entomopathogenic fungi and nematodes show efficacy for biocontrol of apple pests [31,32]. However, little is known about the food webs in these systems and our data provide much needed information on elucidating this important aspect.

In this study we designed a new tool for monitoring plum curculio predation using molecular gut-content analysis. Our analysis of predators showed at least eight different spider families tested positive for feeding on plum curculio in addition to six species of beetles and two species of true bugs. All predators collected and assayed for plum curculio predation were generalist types that may feed on a range of insects. Prior studies suggest that prey abundance drives predation frequency in generalist predators [33]. For instance, prior molecular studies showed that spiders can exhibit this prey switching behavior [34] and confirmed that spiders consume an array of prey species [26]. We are unable to determine the abundance levels of pests because plum curculio are highly elusive and well camouflaged [6]. However, the current trap design does capture plum curculio at a low rates providing an estimate of activity and not a true estimate of abundance. Therefore, we currently cannot link this proportion of predators positive to field infestation level. Nevertheless, our study demonstrated continuous abundance of a diversity of predators in an organic apple orchard and identified a diverse predator assemblage feeding on plum curculio (Table 2).

We identified a diversity of generalist predators positive for plum curculio DNA (Table 2). Potential key predators in the canopy were *Nabis* sp., damsel bugs, *Podisus maculiventris*, the spined soldier bug, and two species of Coccinellidae, lady beetle species. These are all groups of generalist predators known to provide biological control in a range of agricultural systems [16,23,35,36,37]. The finding that some of these predators were consuming plum curculio is interesting. *Nabis* sp. and *Podisus* are both fluid feeders so would need to puncture the bodies of the larvae or the adults to consume plum curculio. The other species have chewing mouth parts so would need to ambush or capture to securing feeding opportunities. This provides evidence that both types of feeding methods can gain access to plum curculio as a prey item and contribute to biological control.

There was also a diversity of epigeal predators that tested positive for the incidence of predation on plum curculio (Table 2). Primarily spiders and some beetles that would more commonly come into contact with pupae on the soil or in the soil. Spiders were the most diverse group observed and are common predatory species in a variety of cropping systems on the soil surface and within soil structures [33,38,39]. Lycosidae, the wolf spiders, in particular may play an important role in biocontrol. These spiders use the soil strata and forage on the surface [23]. Another ground dwelling group that was prevalent in the orchards were the webbuilding spider family, Theridiidae. Larvae dropping from apples or emerging from soil following pupation may get caught in webs. Plum curculio larvae are soft bodied and pupate at 10–60 mm depth, are likely more accessible to the predators we collected when they are closer to the surface. Therefore, enhancing ground predator abundance should increase predation on plum curculio over the season within orchard crops. Cultivating orchard soils in an attempt to disrupt soil-dwelling larval and pupal lifestages was historically recommended as a mechanical plum curculio control tactic [2,40]. These methods could also make these pupae more accessible to epigeal predators and therefore increase encounter rates with epigeal predators. Therefore, the development and application of this molecular approach for orchard systems opens up new questions, and provides a tool to study the effects of cultivation on predators and predation on an economically important pest.

## 5. Conclusions

Using molecular gut-content analysis, a diversity of generalist predators was observed to prey on plum curculio. Although further work is needed on natural enemy effects on plum curculio and specifics on ranking predators best suited to promote biocontrol, our data reveal new information on species that consume this pest. Future work will focus on habitat management to attempt to increase rates of biological control. The integration of nematode based biological control, habitat management to improve epigeal natural enemy populations, and orchard sanitation into a program holds promise for an effective non-chemical strategy to manage this pest. These practices target life-history specific vulnerabilities of this pest in an attempt to disrupt its development. The challenge is that providing habitat for natural enemies and sanitation to remove habitat may be competing tactics. Further research is needed to calibrate the timing and integration of these cultural practices and how these impact biocontrol and pest management.

## Figures and Tables

**Table 1:** Complete list of all predators tested for predation using PCR-assay with plum curculio specific primers. Column for “PCR Positives” indicates number testing positive, and “No. Tested” indicated total number of predators assayed.

Taxonomic Description	PCR Positives	No. Tested
Araneae		
Agelenidae	0	1
Amaurobiidae: *Coras* sp.	0	2
Anyphaenidae	0	4
Araneidae: *Mangora* sp.	0	9
Clubionidae: *Clubiona* sp.	0	6
Corinnidae	0	9
Dictynidae: *Emblyna sp*.	3	8
*Dictyna* sp.	0	3
*Saltonia* sp.	0	2
Unknown-immature	0	4
Linyphiidae: *Agyneta fabra*	2	11
*Erigone* sp.	0	1
*Semljicola* sp.	0	1
Unknown-immature	1	19
Liocranidae: *Agroeca pratensis*	1	2
Lycosidae: *Pardosa* sp.	1	8
*Pirata* sp.	0	30
*Schizocosa* sp.	0	5
*Trabeops* sp.	0	46
*Trochosa* sp.	0	1
Unknown-immature	0	5
Mysmenidae	0	1
Salticidae: *Pelegrina* sp.	1	23
*Hentzia* sp.	0	5
*Metacyrba* sp.	0	1
*Metaphiddipus* sp.	0	5
*Phiddipus* sp.	0	4
Unknown-immature	0	2
Philodromidae: *Philodromus peninsulanus*	1	26
*Tibellus* sp.	0	7
Unknown-immature	0	4
Pisauridae: *Pisaurina* sp.	0	1
Theridiidae: *Enoplognatha ovata*	0	7
*Neottiura* sp.	0	7
*Theridion* sp.	1	32
Unknown-immature	6	37
Thomisidae: *Misumena* sp.	0	1
*Misumenops* sp.	0	3
*Ozyptila* sp.	0	3
*Xysticus* sp.	1	2
Thomisidae: Unknown-immature	1	3
Opiliones: Phalangiidae	0	8
Hemiptera		
Nabidae: *Nabis* sp.	7	32
Pentatomidae: *Podisus maculiventris*	2	5
Coleoptera		
Cantharidae: *Podabrus* sp.	1	8
Carabidae: *Amara* sp.	1	4
*Anisodactylus* sp.	0	2
*Bradycellus* sp.	0	3
*Colliuris* sp.	0	1
*Harpalus* sp.	1	2
Coccinellidae: *Brachiacantha ursina*	2	3
*Coleomegilla maculata*	0	7
*Harmonia axyridis*	3	4
*Propylea quatuordecimpunctata*	0	2
Staphylinidae: *Stenus* sp.	2	6

**Table 2:** This table highlights the predator species that tested positive for plum curculio using species-specific primers. Predators determined to have recently fed on plum curculio are indicated with the number of positive PCRs for each predatory taxon. The total number tested is indicated by No. tested. For total list of predators tested see Table 1.

Taxonomic Description	PCR Positives	No. Tested
Araneae		
Dictynidae: *Emblyna sp*.	3	8
Linyphiidae: *Agyneta fabra*	2	11
Liocranidae: *Agroeca pratensis*	1	2
Lycosidae: *Pardosa* sp.	1	8
Salticidae: *Pelegrina* sp.	1	23
Philodromidae: *Philodromus peninsulanus*	1	26
Theridiidae (immature)	7	69
Thomisidae (immature)	2	5
Hemiptera		
Nabidae: *Nabis* sp.	7	32
Pentatomidae: *Podisus maculiventris*	2	5
Coleoptera		
Cantharidae: *Podabrus* sp.	1	8
Carabidae: *Amara* sp.	1	4
*Harpalus* sp.	1	2
Coccinellidae: *Brachiacantha ursina*	2	3
*Harmonia axyridis*	3	4
Staphylinidae: *Stenus* sp.	2	6
